# 

**Published:** 1888-05

**Authors:** 


### CONTENTS:

ARTICLE I.—Treatment of Pain. By James Maughan, M. D ........... 1

" II.—The Combination of Metals for Filling Teeth. By Dr. C. T. Tockwell ........... ............. 8

" III.—Unhealthy Breath. By Alfred H. Tester, L. D. S ........ 16

" IV.—Pyorrhœa Alveolaris with Practical Cases. By J. L. Gish, M. D., D. D. S ....................... 23

" V.—Odontological Society...... ............................ ........ 31

### MONTHLY SUMMARY.

Former Illiberality in the Profession...... .... ........................... ..... 43

Waste.............................................. ............ ............ ...... 44

Make Your Work a Pleasure................. ... .................. ........... 45

Cleaning the Teeth ......... ............. ....................................... ......... 46

Sweets............ ................................................................ 47

The Philosophy of a Cold................................................. 47

In Favor of a Vegetable Diet .. .............. ................................ ...... 48

Mercury .............................................................. ............. 48

